# Ligation of the Carotid Artery1Read before the Montreal Veterinary Medical Association.

**Published:** 1896-03

**Authors:** E. H. Morris


### LIGATION OF THE CAROTID ARTERY.[1]

BY E. H. MORRIS.

THE subject was a two-year-old colt, well bred, in good condition, and of a highly nervous temperament. He had been tied near an iron picket fence, and in his restiveness tried to jump over it. The hitch rein being still tied caused him to fall on the fence, severely lacerating the deeper tissues of the neck at the lower third of the cervical vertebra, causing much hemorrhage. I was called to see the colt, but not until after every farmer who happened to be present had exhausted all his *sure* methods of arresting hemorrhage. Some had stuffed dried leaves and dirty rags in the wound, others had packed coal ashes in it, and some had even tied strings around the animal's tail to stop hemorrhage of the carotid.

When I arrived I found the animal so weak that he could hardly stand, and he was easily thrown down by pulling his head to one side and twisting it a little. After having carefully picked out all the dirt and trash I washed the wound carefully, and was able to locate the exact source from which the blood was coming. The wound, which was on the right side, was about six inches long, running in a longitudinal direction through the scalenus muscle, exposing at least four inches of the trachea, carotid artery, and vagus nerve. On observing more closely a torn place in the carotid, about one-quarter of an inch in length, could easily be seen, from which the blood was oozing.

I had never heard of the carotid being ligatured, and at first was puzzled to know what was to be done. The first thought that struck me was, How was this side of the head and face to receive nourishment? The animal was bleeding to death, and

[1] Read before the Montreal Veterinary Medical Association.

what was to be done was to be done immediately, as the pulse was already imperceptible at the submaxillary artery. I applied a strong silk ligature below the wound in the vessel, and through fear of this giving way applied another at the same place. I also put one beyond the wound; the peripheral ligature was necessary on account of the anastomosis through the vertebral into the occipital and innominate arteries.

We next washed the wound out with a carbolic solution and put eight or ten sutures in the muscle and as many in the skin, leaving an opening at the bottom of the wound for pus to escape if any should form. He was then allowed to rise, which he did with much exertion. He was so weak that he could not walk without staggering from one side to another, and would occasionally plunge forward, seemingly in a fainting or unconscious manner, into the crowd which had gathered. It was impossible to keep him quiet. Next he was given an ounce of whiskey subcutaneously, which seemed to bring him more to his senses, and he was removed to the stable, where an iodoform dressing was applied. At intervals of about three or four hours he was brought out of his box, and cold water was allowed to run over the wound for an hour or two at a time. This was to keep down the inflammation and prevent arteritis being set up. He was taken to the stable about six o'clock in the afternoon. About 9 P.M. I tried to give him a stimulating drench, when he gave a lunge and fell with his head under the manger. We concluded that worrying him by drenching would do more harm than the drench would do good, as he was very wild. We did nothing more with him till morning. Next morning the pulse was imperceptible on the injured side, but on the other side was very weak and rapid. Temperature was 98° F. and the right side of the face and the right ear were much cooler than the left, the right carotid being the ligatured one. The wound looked well and the sutures were still in good condition, much to my surprise, for I fully expected them to have been torn out. There was no great swelling and no exudate, but it was very hot and painful, so I continued the cold-water treatment.

The animal ate some green grass and drank heartily, but was still very weak, and I continued through the day to administer mild stimulants. For the next three days we kept up the cold-water treatment and tried to keep the wound aseptic. He was much stronger, the appetite returning. The owner, who lived fourteen miles in the country, came in on the fourth day after the injury and took the animal home against my advice, for I thought the colt was too weak to make such a long trip. However, he did stand it very well, and the owner told me that the wound healed entirely, without any appreciable slough, and the sutures kept in place to the last.

The reason the sutures held so well in this case is that the wound was so painful that he could not rub against anything without causing much discomfort. I have not seen the animal since he was taken home, but the owner told me that there was not the slightest scar left, and that one side of his face was as warm as the other, and the colt seemed as well as he ever was.

